# Understanding the structure and dynamics of anti-inflammatory corticosteroid dexamethasone by solid state NMR spectroscopy

**DOI:** 10.1039/d0ra05474g

**Published:** 2020-10-12

**Authors:** Krishna Kishor Dey, Manasi Ghosh

**Affiliations:** Department of Physics, Dr. Harisingh Gour Central University Sagar-470003 Madhya-Pradesh India; Physics Section, MMV, Banaras Hindu University Varanasi-221005 Uttar-Pradesh India manasi.ghosh@bhu.ac.in

## Abstract

For decades corticosteroid dexamethasone has been applied as an anti-inflammatory, immunosuppressant, and decongestant, in the prevention of postoperative nausea and vomiting (PONV), and for auto-immune diseases, allergic reactions, total hip arthroplasty (THA), and cancer. Recently *in vitro* studies suggested that it may be beneficial to deal with the COVID-19 pandemic. This important drug molecule was investigated by solid state NMR measurements to provide more complete features of its structure and dynamics at atomic scale resolution. The spin–lattice relaxation time at twenty-two different carbon sites of dexamethasone was determined by the Torchia CP method. The principle components of the chemical shift anisotropy tensor were determined by ^13^C two-dimensional phase adjusted spinning sideband (2DPASS) cross-polarization magic angle spinning (CP-MAS) solid state NMR experiments. The molecular correlation time at twenty-two crystallographically different carbon sites of dexamethasone was calculated by considering that the spin–lattice relaxation mechanism of the ^13^C nucleus is mainly governed by the chemical shift anisotropy interaction and the heteronuclear dipole–dipole coupling. The spin–lattice relaxation time of carbon nuclei resides on ‘A’, ‘B’, ‘C’, and ‘D’ rings and the side-chain of dexamethasone is quite large, which implies the close-packed arrangement of the molecule. The difference in molecular correlation time at various regions of the molecule demonstrates the existence of different degrees of freedom within the molecule. This may be the reason for the various biological activities exhibited by the molecule. These types of detailed features of the structure and dynamics of such an important drug with multiple biological activities are necessary to develop the advanced medicine and it will also help to understand the structure–activity relationships of corticosteroid.

## Abstract 2

The structure and dynamics of dexamethasone is determined by measuring CSA tensor, site-specific spin–lattice relaxation time.

## Graphical abstract

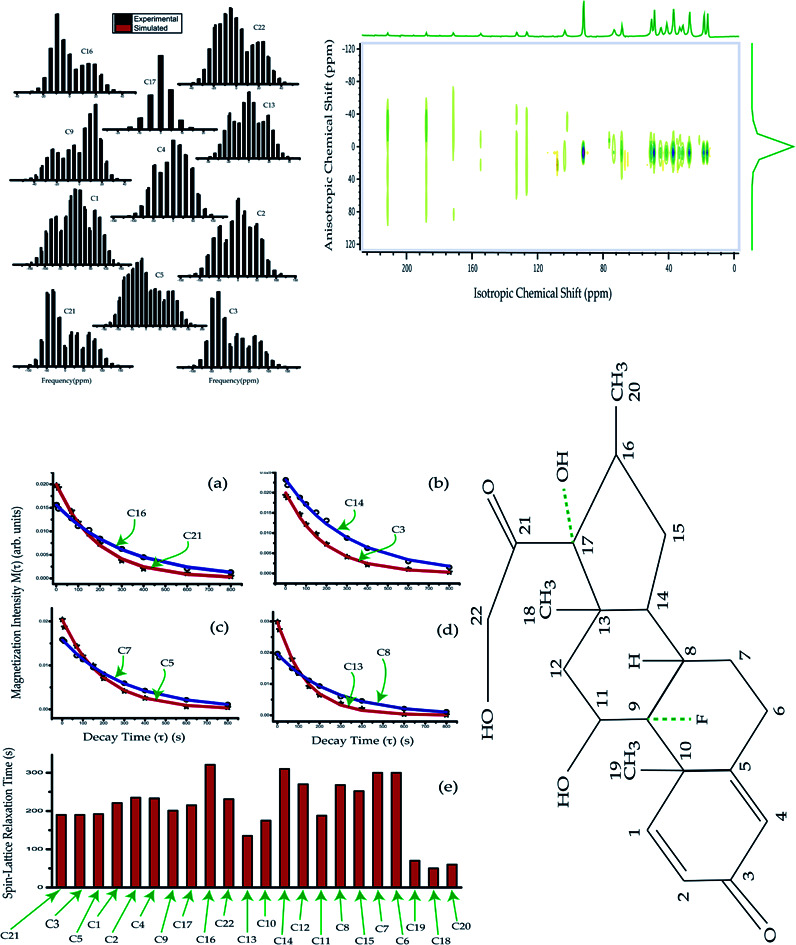

## 1. Introduction

The drug dexamethasone has been used as an anti-inflammatory, immunosuppressant, and decongestant, in the prevention of postoperative nausea and vomiting (PONV), and for auto-immune diseases, allergic reactions and cancer.^1,2^*In vitro* studies suggested that dexamethasone, (which controls inflammation by inhibiting peripheral phospholipase), may be beneficial to reduce the death rate of the COVID-19 pandemic, caused by respiratory syndrome coronavirus 2 (SARS-CoV-2) infection.^3^ Because of its antiemetic properties, dexamethasone is administered to patients undergoing highly emetogenic chemotherapy.^4^ This corticosteroid exercises its antiemetic activity *via* prostaglandin antagonism.^5^ Dexamethasone is also used to manage vomiting during chemotherapy.^6^ There are several reasons for using a combination of dexamethasone and 5-HT_3_ receptor antagonist for the control of chemotherapy-induced nausea and vomiting.^7,8^ One of the reasons is that the corticosteroids diminish the levels of 5-hydroxytryptophan in neural tissue by depleting its precursor tryptophan.^9^ The anti-inflammatory properties of corticosteroids hinder the discharge of serotonin in the gut.^10^ In comparison with other antiemetics, dexamethasone has the prospective to excite the pharmacological receptor.^11,12^ The amalgamation of dexamethasone with ondansetron or granisetron further reduced the risk of PONV.^12^ Dexamethasone is such a corticosteroid which is used to serve multiple purposes like it regulates energy metabolism and releases energy substrates by enhancing hepatic gluconeogenesis, plummet glucose utilization, boost muscle protein catabolism and lipolysis,^13^ regulates catecholamine levels,^14^ inhibit catechol-*O*-methyl transferase (COMT),^15^ activates monoamine oxidase (MAO),^16^ protects cell death,^17–19^ intensify the appearance of the serotonin transporter (5-HTT)^20,21^*etc.*

The biological effects of corticosteroid hormones are resolute by their influence on the protein synthesis rate in target tissues. After entering the target cell, the corticosteroid combines with the cytoplasmic receptor protein. After that, the hormone–receptor complex proceeds towards the nucleus, where it connects with the acceptor site on the genome and induces RNA species. The mechanism through which the hormone–receptor complex binds with the acceptor site is indefinite. The role of a corticosteroid hormone is determined by its ability to make a conformational change in the receptor protein so that the protein binds with chromatin.^22^

The most significant structural features of a corticosteroid are concerned about the flexibility of the unsaturated nucleus, the restrictions on the flexibility of side chains, and the existence of fascinating characteristic patterns subtended by inter-helical and inter-helical hydrogen bonds. The main goal of the current work is to pursue a molecular level elucidation of structure and dynamics of corticosteroid dexamethasone by determining CSA-parameters, spinning CSA sideband pattern, spin–lattice relaxation time and molecular correlation time at twenty two crystallographically different carbon sites of the molecule. The CSA parameters are determined by the two-dimensional phase adjusted spinning side-band (2DPASS) cross-polarization (CP) magic angle spinning (MAS) SSNMR experiment.^23,24^ The site specific spin–lattice relaxation time is determined by the Torchia CP method.^25^ The CSA parameters can be also determined by – two dimensional MAS/CSA NMR experiment;^26^ SUPER (separation of undistorted powder patterns by effortless recoupling);^27^ ROCSA (recoupling of chemical shift anisotropy);^28^ RNCSA (γ-encoded RN^ν^_n_-symmetry based chemical shift anisotropy);^29^ 2DMAF (two-dimensional magic angle flipping) experiment;^30–32^ 2DMAT (two-dimensional magic angle turning) experiment.^33^ 2DPASS CP-MAS SSNMR technique was applied to determine the structure and dynamics of biopolymers, biomedicine.^34–40^ CSA parameters and spin–lattice relaxation time are the most important NMR parameters measured from high-resolution NMR experiments for exploiting the structure and dynamics of a molecule at atomic level resolution. CSA tensor offers information about the local symmetry of the electronic distribution surrounding the nucleus. The spin–lattice relaxation mechanism of carbon nuclei is mainly governed by chemical shift anisotropy interaction and hetero-nuclei dipole–dipole coupling. Hence principal components of CSA parameters not only furnish the information about the local three-dimensional structure surrounding the nucleus, but it also dispenses information about the molecular dynamics. The detail features about the structure and dynamics of such an important drug which has the prospective to control COVID-19 pandemic will enlighten the path of inventing advanced medicine.

## 2. Experimental

### 2.1 NMR-measurements

The active pharmaceutical ingredient dexamethasone was purchased from Sigma Aldrich. ^13^C CP-MAS solid state NMR experiments were performed on a JEOL ECX 500 NMR Spectrometer, operating at resonance frequency 125.721 MHz. The spectrometer was well equipped with a 3.2 mm JEOL double resonance MAS probe. The mass speed for ^13^C CP-MAS NMR experiment was 10 kHz. Contact time for Cross-Polarization (CP) was 2 ms, with repetition interval 30 seconds, and SPINAL-64 ^1^H decoupling at 3072 accumulations time. ^13^C-spin–lattice relaxation experiment was performed by using Torchia CP method with contact time 2 ms.^25^

### 2.2 CSA-measurements

The information regarding the three-dimensional molecular conformation and electron distribution is lost in the isotropic spectrum obtained by ^13^C-CP-MAS-SSNMR experiment because all these information are encoded in anisotropic interactions. The chemical shift anisotropy (CSA) information can be restored by reducing the magic angle spinning (MAS) frequency less than the span of the chemical shift anisotropy. When the MAS speed is less than the span of CSA, the solid state NMR spectrum is flanked into equally spaced sidebands and the spacing between two sidebands is equal to the MAS frequency.^41,42^ The chemical shift anisotropy (CSA) parameters can be extracted by using the intensities of these spinning sidebands by incorporating Herzfeld and Berger method.^43^

At low MAS frequency, the CSA parameters can be extracted by two-dimensional magic angle flipping (2DMAF) experiment,^30,31^ two-dimensional magic angle turning (2DMAT) experiment,^33^ and 2DPASS.^23,24^ But 2DMAF experiment cannot be performed in a commercial probe. It is necessary to design a complicated probe, by which cone can achieve the flipping of the magic angle during one experiment. As 2DMAT is not a constant time experiment, so it would be difficult to extract CSA information from the two-dimensional spectrum.

The pulse sequence of the 2DPASS experiment with five π pulses was introduced by Antzutkin *et al.*^23^ The time duration of the PASS sequence is constant and the time gap among five π pulses varied according to the PASS equations. The 2DPASS CP MAS SSNMR experiments were carried out at two MAS frequencies 600 Hz and 2000 Hz. The contact time was 2 ms to maintain CP condition. For ^13^C nucleus 90 degree pulse length was 3 μs.

## 3. Results and discussion

### 3.1 Solid state NMR spectral analysis

**Fig. 1:**
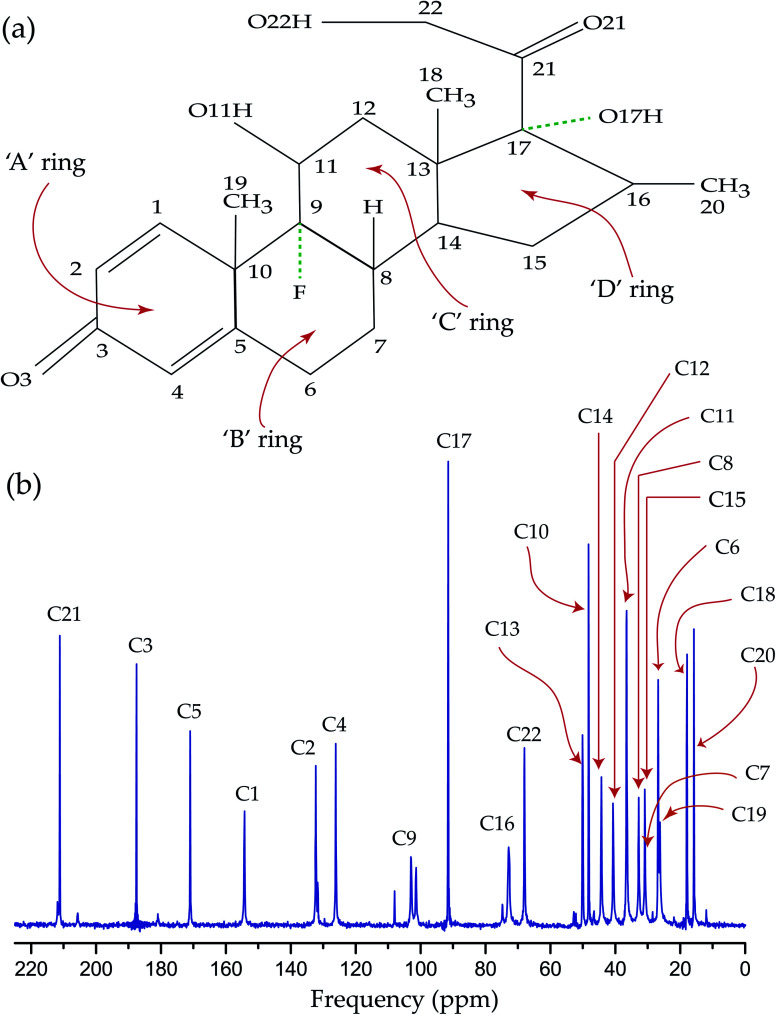
(a) Structure of dexamethasone, (b) ^13^C CP-MAS SSNMR spectrum of dexamethasone.

**Fig. 2:**
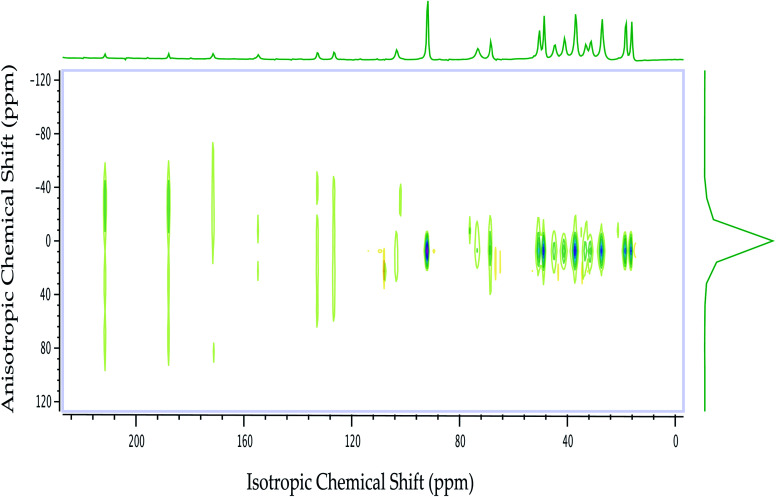
^13^C 2DPASS CP-MAS SSNMR spectrum of dexamethasone. The direct dimension represents isotropic chemical shift, and the indirect dimension represents anisotropic chemical shift.

The synthetic corticosteroid dexamethasone (C_22_H_29_O_6_F) is formed by twenty two carbon atoms, five oxygen atoms, four rings, and one fluorine atom also known as 1-dehydro-9α-fluoro-16α-methylhydrocortisone or as 9α-fluoro-11β,17α,21-trihydroxy-16α-methylpregna-1,4-diene-3,20-dione. Two among five oxygen atoms are bonded with C21 and C3 carbon atoms as ketone carbonyls and the other three reside on hydroxyl groups attached with C11, C17, and C22 carbon atoms. The fluorine atom, bonded with C9 carbon atom, resides at the junction of two rings. Most of the corticosteroid is fabricated by three six-membered rings ‘A’, ‘B’, and ‘C’, one five membered ring ‘D’, and a flexible side chain/functional group attached with C17 carbon. Fig. 1(a) shows the chemical structure of dexamethasone and Fig. 2(b) represents ^13^C CP-MAS SSNMR spectrum of dexamethasone at MAS frequency 10 kHz. All the resonance lines are assigned by following the book of Jeffrey H. Simson.^44^Table 1 represents the isotropic chemical shift of various chemical groups of dexamethasone. Fig. 1(a) shows that the C21 and C3 atoms are double bonded with a electronegative oxygen atom. The presence of electronegative atom attracts the electron cloud of the nearby atoms. As a result, the nuclear shielding effect is decreased and the effective magnetic field experienced by the nuclei is increased. That's why the isotropic chemical shift of C21 and C3 nuclei are the largest among all other carbon nuclei of dexamethasone. In the next section, it will be discussed that not only the isotropic chemical shift but the values of principal components of anisotropic chemical shift tensor are also very high for those two carbon nuclei.

**Table 1:** Position of chemical shift of various groups of the dexamethasone molecule by ^13^C CP-MAS spectral analysis

Carbon atom	Position of isotropic chemical shift (ppm)	Group type
C21	211.5	Nonprotonated
C3	187.8	Nonprotonated
C5	171	Nonprotonated
C1	155	Methine
C2	132.4	Methine
C4	126.2	Methine
C9	103/101 (splitting is observed because of *J* coupling)	Nonprotonated
C17	91.7	Nonprotonated
C16	73.2	Methine
C22	68.1	Methylene
C13	50.7	Nonprotonated
C10	48.2	Nonprotonated
C14	44.8	Methine
C12	41	Methylene
C11	36.6	Methine
C8	33.2	Methine
C15	31.5	Methylene
C7	30.8	Methylene
C6	27.1	Methylene
C19	26.4	Methyl
C18	18.5	Methyl
C20	16.1	Methyl

Fig. 1(b) shows that the resonance line corresponds to C9 nuclei (attached with fluorine atom) is split due to *J*-coupling as the ^13^C CP-MAS SSNMR spectrum is recorded with ^1^H decoupling (without ^19^F decoupling). The isotropic chemical shift of methyl groups C18, C19, and C20, which uplifted the potency and duration of drug action, are lowest. The isotropic chemical shift of C1, C2, C4, C5 atoms reside on the ‘A’ are quite large. The flexible side chain attached with C17 atom and the hydroxyl group connected with C11 atoms is involved in protein interaction. The active corticosteroid hormones contain unsaturated bonds, which is responsible for conformational flexibility. The unsaturated ‘A’ ring is the area of the conformational flexibility of the corticosteroid molecule. The nature of the flexibility of the unsaturated ‘A’ ring depends upon the conformation of the ring – it may form 2β-sofa; 1α,2β-half-chair; and 1α-sofa.^22^ If the five atoms of ‘A’ ring are coplanar and the sixth one is out of the plane, then this conformation is described as 1α-sofa conformation. It is described as 2β-sofa conformation, when the sixth atom is on the α-side.^22^ On the other hand, if four adjacent atoms of the ‘A’ ring are coplanar and the remaining two are equally displaced on two opposite sides of the plane, then it is called 1α,2β-half-chair conformation.^22^ The additional substituents on the ‘A’ ring shift the ring conformation towards the ideal half-chair or sofa forms. For dexamethasone the ‘A’ ring takes 1α,2β-half-chair conformation and the fluorine substitution with C9 atom stabilize the 1α,2β-half-chair conformation.^22^

### 3.2 Determination of the principal components of CSA tensor

The effective magnetic field experienced (*B*_eff_ = (1 ± *δ*)*B*_ext_) by the nucleus is not exactly equal to the applied magnetic field, sometimes it is greater or lower than the external magnetic field due to the intervention of electron cloud surrounding the nucleus. The electrons revolve around the nucleus in presence of external magnetic field, induced a secondary magnetic field, whose magnitude is directly proportional to the external magnetic field. As a consequence, the nuclear Larmor precession frequency is getting altered – this effect is known as chemical shift. The values of chemical shift depend on the direction of molecular orientation and molecular conformation. It is a second-rank tensor. It has nine components. It is known as chemical shift anisotropy (CSA) tensor. Chemical shift anisotropy interaction plays a paramount important role in ^13^C spin–lattice relaxation mechanism. Hence, CSA tensor not only encoded the information about the molecular conformation and electronic surrounding, but it also can provide valuable information about the nuclear spin-dynamics. In principal axis system (PAS) all the off-diagonal terms of CSA tensor are averaged out, only three diagonal parameters are survived, which are known as principal components of CSA tensor. In liquid state NMR spectroscopy, due to the continuous tumbling motion of the molecule all the anisotropic interactions are averaged out.

Table 2 shows the principal components of the chemical shift anisotropy tensor of dexamethasone. The chemical shift anisotropy parameters of carbonyl group carbon C21, C3 are substantially large due to the proximity of electronegative oxygen atom. The electronegative atom attracts the electron cloud surrounding the carbon-nucleus. As a consequence, the effective magnetic field experienced by the carbon nucleus is increased due to the deshielding effect. This is one of the reasons for the higher values of chemical shift. Another reason is the presence of hydrogen bonding with the oxygen atoms attached to C21 and C3 atoms. The close pack arrangement and the greatest degree of conformational flexibility of the corticosteroid are maintained due to the presence of hydrogen bonding. Hydrogen bonds induced polarization on an electron cloud of neighbouring atoms. As a result, there arises a distortion in the spherically symmetric charge distribution surrounding the carbon nucleus and there induced a magnetic anisotropy^45^

1 *δ*_anis_ = {Δ*X*_|_(3 cos^2^ *θ*_1_ − 1) + Δ*X*_⊥_(3 cos^2^ *θ*_2_ − 1)}/3*R*^3^

where *θ*_1_ and *θ*_2_ are the angle subtended by the radius vector with *x*-axis and *z*-axis respectively. Where Δ*X*_|_ = *X*_*z*_ − *X*_*x*_ and Δ*X*_⊥_ = *X*_*y*_ − *X*_*x*_ represent the susceptibility parallel and perpendicular to the applied magnetic field respectively. The higher degree of directional specificity leads to the higher values of CSA parameters for C3 and C21 nuclei.

**Table 2:** Chemical shift anisotropy parameters of ^13^C carbon nuclei of dexamethasone at chemically different carbon site

CSA parameters of dexamethasone								
Carbon from different chemical environment	*δ*_11_ (ppm)	*δ*_22_ (ppm)	*δ*_33_ (ppm)	Span (ppm)	Skew	*δ*_iso_ (ppm)	Anisotropy (ppm)	Asymmetry
C21	325.5	165.8	143.1	182.4	−0.7	211.4	171.1	0.2
C3	300.3	145.2	118	182.3	−0.7	187.8	168.7	0.2
C5	309.1	143.3	61.9	247.2	−0.3	171.4	206.5	0.6
C1	269.1	161.2	34.7	234.3	0.1	155	−180.4	0.9
C2	218.5	137.7	40.9	177.6	0.1	132.4	−137.2	0.9
C4	198.9	132.9	47	151.9	0.1	126.3	−118.9	0.8
C9	127.7	115.1	66.2	61.5	0.6	103	−55.2	0.3
C17	101.2	92.5	81.4	19.8	0.1	91.7	−15.5	0.8
C16	102.3	63.6	58.8	48.5	−0.6	73.2	43.7	0.3
C22	103.2	62.1	38	64.3	−0.3	68.1	52.7	0.6
C13	81.4	50.6	19.9	61.4	0	50.6	46.1	1
C10	65.8	52.6	26.4	39.4	0.3	48.3	−32.8	0.6
C14	88	27.9	18.6	69.4	−0.7	44.8	64.8	0.2
C12	54.1	39.7	29.4	24.7	−0.2	41.1	19.5	0.8
C11	55.6	39.3	15	40.6	0.2	36.6	−32.4	0.7
C8	56.2	35.1	8.3	47.9	0.1	33.2	−37.4	0.8
C15	51.3	32.7	10.5	40.8	0.1	31.5	−31.5	0.9
C7	44.5	30.8	17.1	27.3	0	30.8	−20.5	1
C6	45.5	24	11.6	33.9	−0.3	27	27.7	0.7
C19	44.7	24.3	10.1	34.6	−0.2	26.4	27.5	0.8
C18	30	16.3	9.1	20.9	−0.3	18.5	17.3	0.6
C20	28.5	15.3	4.5	24	−0.1	16.1	18.6	0.9

The double bonds between C1 and C2 and C4 and C5 of the six membered ‘A’ ring increased the anti-inflammatory activity and diminished the salt-retaining properties of dexamethasone. The CSA parameters of those nuclei are significantly high compared to other carbon nuclei reside on ‘B’, ‘C’, and ‘D’ rings due to the presence of π-electrons. The existence of the fluorine atom, bonded with C9 carbon atom, increases the binding affinity of the corticosteroid receptor and retarded oxidation of the hydroxyl group attached with C11 atom. The anti-inflammatory assays and glycogen deposition rates are enhanced by the presence of fluorine atom.^22,46–49^ In general, the biological activity of corticosteroid is proportionally increased with the electronegativity of the C9 substituent. The CSA parameters of C9 nuclei are higher than C6, C7, C18, and C10 nuclei reside on the ‘B’ ring. The electronegative fluorine atom attracts the electron cloud surrounding C9 nuclei. As a consequence, the influence of the nuclear shielding effect is lowered and the effective magnetic field experienced by the nucleus is increased, which leads to the higher values of the CSA parameters.

The conformation of ‘A’ ring as well as the distance of O3–O11 (6.822 Å), O11–O17 (5.321 Å), and the O3-mean plane C5–C17 (2.57 Å) are prime factors on which the activity of glucocorticoids depend on.^46–48^ The potency of the drug increases when these distances are larger. The CSA parameters of all the carbon nuclei reside on ‘A’ ring are larger except C10. C10 is in sp^3^ hybridization state, which is the reason of the lower values of CSA parameters of C10 nuclei. The anti-inflammatory activity increases when the ‘A’ ring deviates more from the C5–C17 mean plane. The bonding of the fluorine atom with C9 is the reason of the deviation in the orientation of the ‘A’ ring with respect to the plane of ‘B’, ‘C’, and ‘D’ rings. The greatest conformational variations are observed on unsaturated ‘A’ ring and C17 flexible side-chain.^22^

**Fig. 3:**
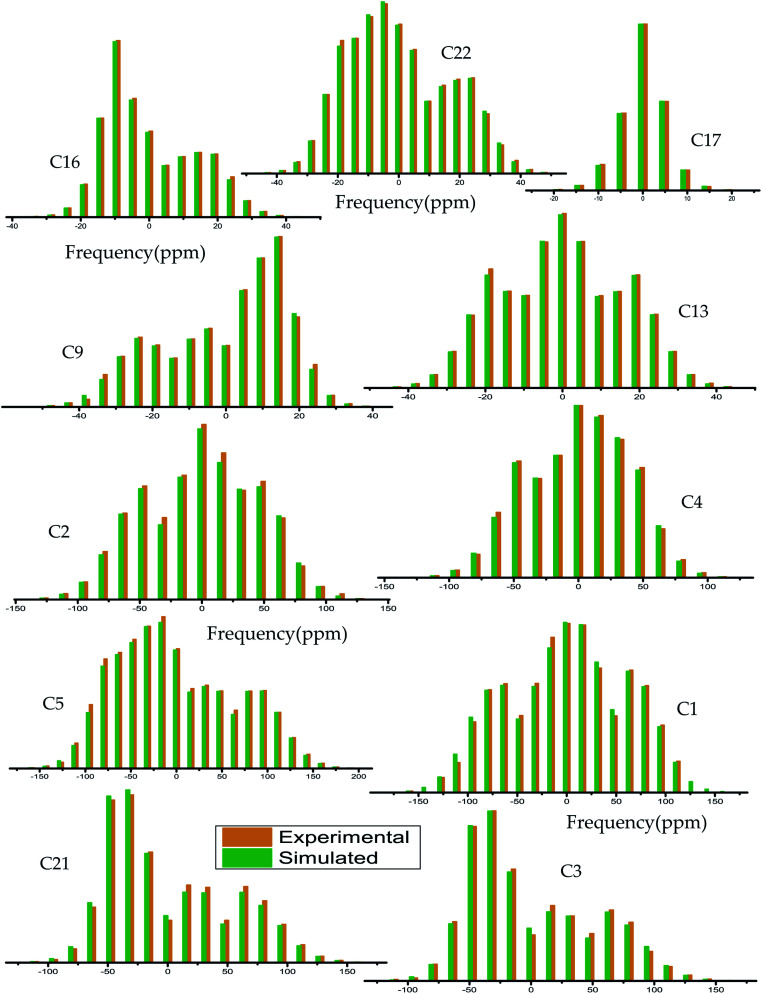
Spinning CSA side-band pattern at crystallographically different carbon nuclei sites of dexamethasone.

**Fig. 4:**
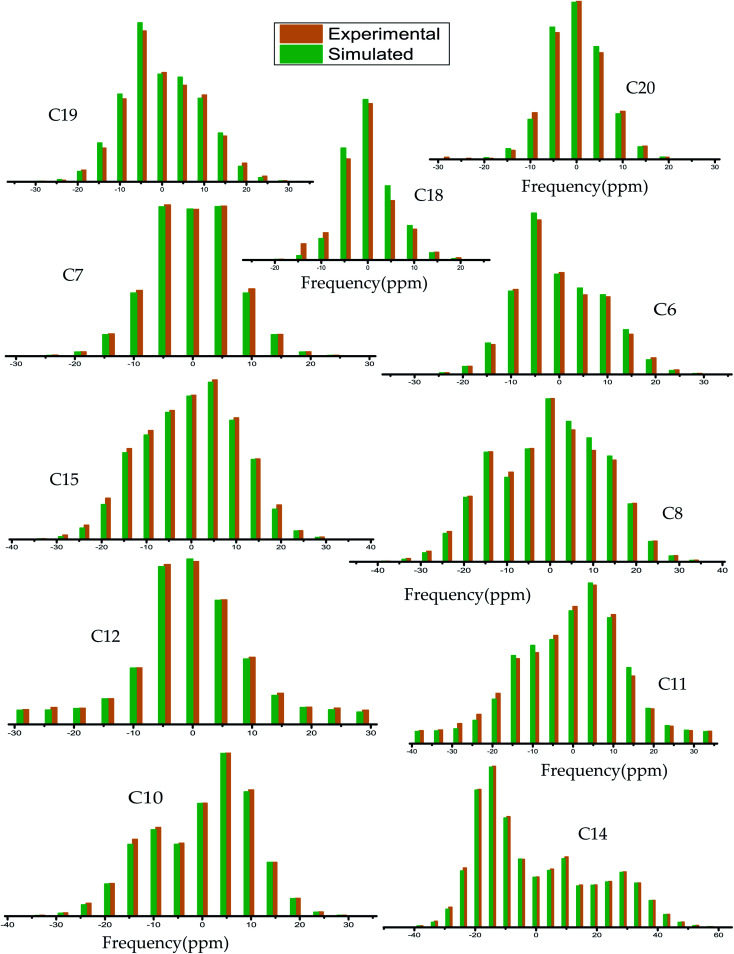
Spinning CSA side-band pattern at crystallographically different carbon nuclei sites of dexamethasone.

**Fig. 5:**
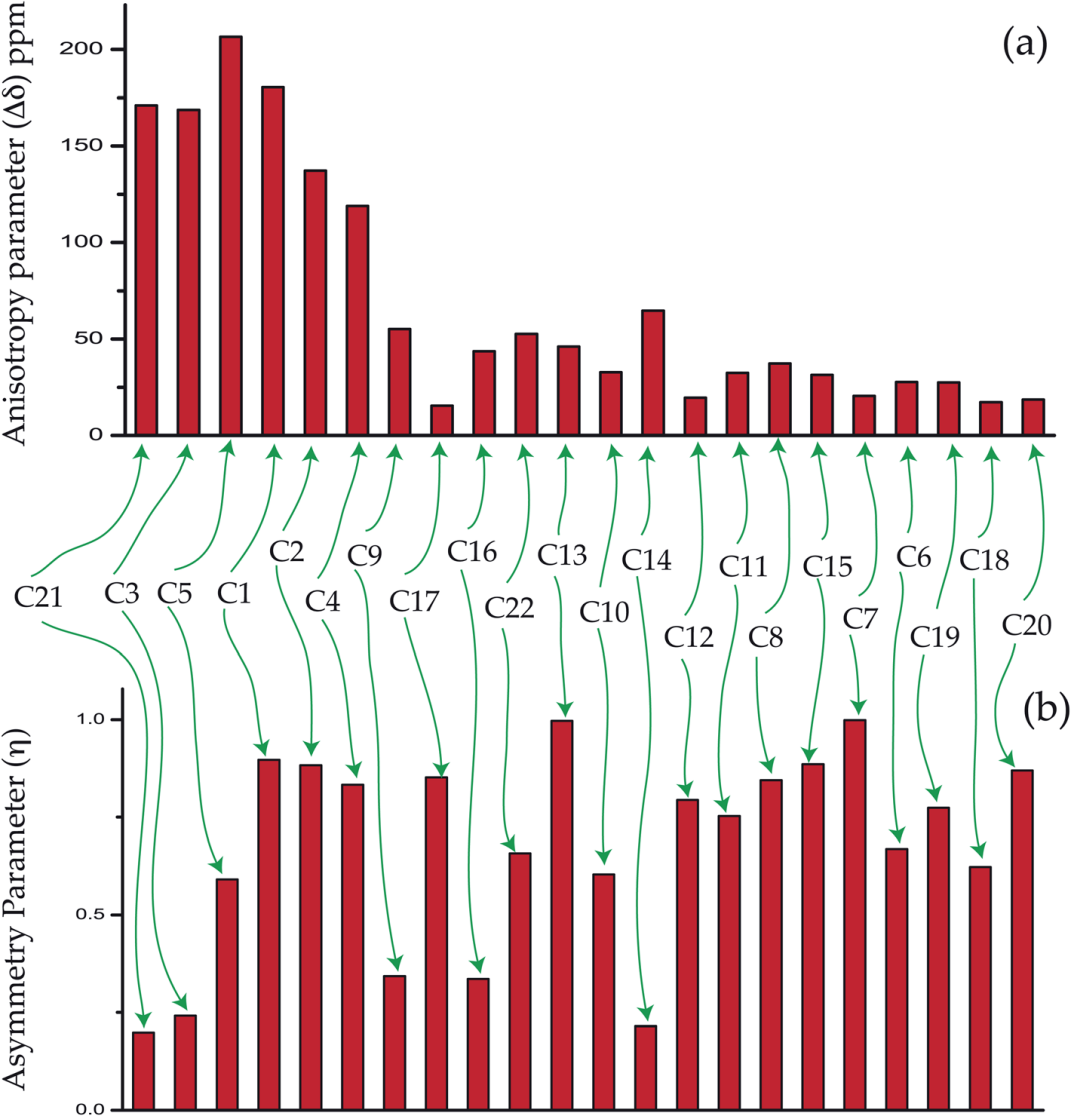
The bar-diagram of (a) anisotropy parameter and (b) asymmetry parameter of dexamethasone at crystallographically different sites.

The magnitude of anisotropy parameter (Δ*δ* = *δ*_33_ − (*δ*_11_ + *δ*_22_)/2) measures the largest separation of spinning CSA sideband pattern from the center of gravity (*δ*_iso_ = (*δ*_11_ + *δ*_22_ + *δ*_33_)/3). The sign of the anisotropy parameters says on which side of the center of gravity, the separation is largest. Table 2 says that the anisotropy parameters of C21, C1, C2, C3, C4, and C5 carbon nuclei are the largest among all the carbon atoms of dexamethasone. Fig. 3 and 4 show the spinning CSA sideband pattern of twenty-two carbon nuclei of dexamethasone. The different CSA pattern at various sites of the molecule implies that the molecular conformation and electronic distribution are different surrounding the twenty-two carbon nuclei. The asymmetry parameter is defined as *η* = (*δ*_22_ − *δ*_11_)/(*δ*_33_ − *δ*_iso_). If *δ*_22_ = *δ*_11_ or *δ*_22_ = *δ*_33_, then the spinning CSA side-band pattern is axially symmetric. If the value of *η* ≤ 0.3, the CSA pattern is nearly axially symmetric. On the contrary, when *η* ≥ 0.8, the CSA pattern is highly asymmetric. From Table 2 and Fig. 5(b), it is clear that the spinning CSA sideband patterns are nearly axially symmetric for C3, C9, C14, C16 and C21 nuclei and it is highly asymmetric for C1, C2, C4, C7, C8, C12, C13, C15, C17, C18, C20 nuclei. Hence, the asymmetry parameter basically measures how much the spinning CSA side-band pattern deviates from its axially symmetric shape. Skew 
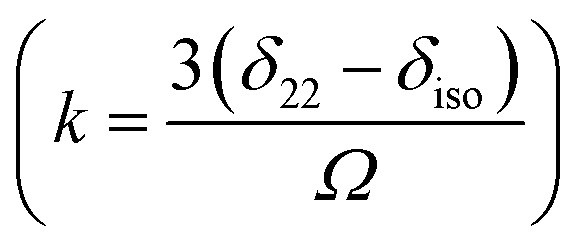
 represents the orientation of the asymmetric pattern.

The substituent of the flexible group at C17 also plays significant role in glucocorticoid activity.^22,46–49^ The ‘span’ and ‘anisotropy’ parameters of C17 nuclei are low compared to other nuclei on ‘D’ ring. The flexible side chain is attached with C17 nuclei – this may be the reason of lower values of CSA parameters.

### 3.3 Determination of spin–lattice relaxation time and molecular correlation time

The spin–lattice relaxation time of dexamethasone varies from 321 s to 50 s. The spin–lattice relaxation time and molecular correlation time of methyl group carbon nuclei C18, C19, C20 are much lower than those carbon atoms reside on six-membered rings ‘A’, ‘B’, ‘C’ and five membered ring ‘D’. Methyl groups increase the potency and duration of drug action. The spin–lattice relaxation time of the carbon atoms reside on the rings of the molecule is substantially high because of the robust structure of the molecule incorporated by hydrogen bonding. In corticosteroid structure, the water molecule, hydroxyl (OH) and C=O substituents fabricate a hydrogen bond network. The twisting of the ‘A’ ring away from the C5–C17 mean plane allows the molecule to grasp the fascinating packing arrangement of long helices organized by the water molecule. Two hydrogen bonds attached with O11 and O22 atoms connect the water molecule with the corticosteroid. Another intra-helical hydrogen bond connects the beginning and end part of the molecule, which enables the molecule to take a helical configuration. The inter-helical hydrogen bonds stabilized the robust helical structure of the molecule.

The corticosteroid hormones interact with receptor proteins through the formation of hydrogen bonds involving the oxygenated functional groups. The higher degree of directional specificity is observed at the position of hydrogen bond donors and acceptors.^22^ The hydrogen bonds associated with the hydroxyl group attached with C11 atom is bonded *trans* to the C9–C11 bond. The hydrogen bonds associated with the hydroxyl group attached with C17 atom is bonded *trans* to the C13–C17 bond. The directionality of hydrogen bond donors and acceptors emerge a fascinating crystallographic pattern for corticosteroid. The crystal packing arrangement is controlled by intermolecular and intramolecular forces.^22^

**Fig. 6:**
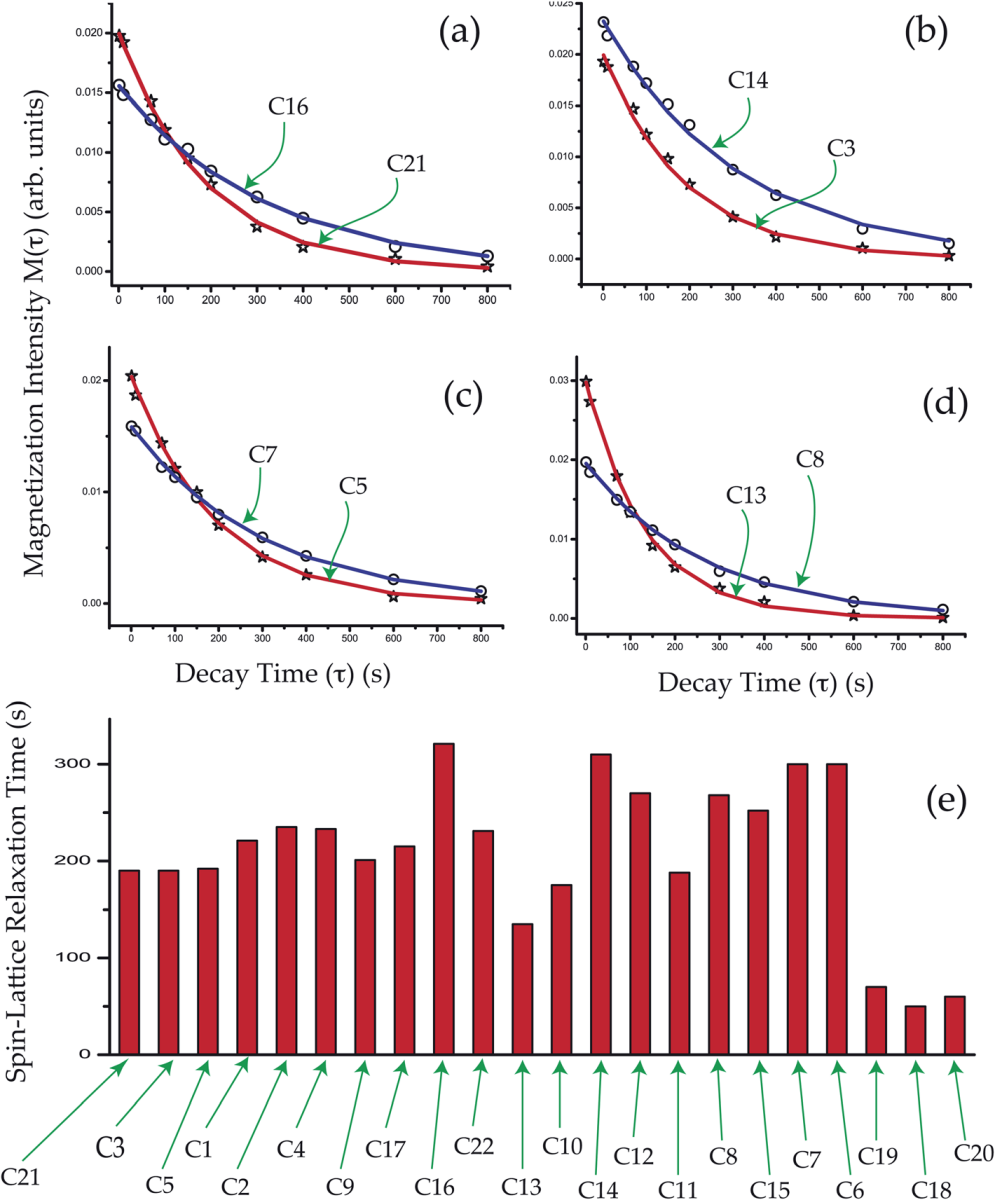
(a–d) Magnetization decay curves of dexamethasone at crystallographically different carbon nuclei sites. (e) Spin–lattice relaxation time of dexamethasone at crystallographically different carbon nuclei sites.

The crystallographic findings demonstrate that the orientation of the side-chain attached with C17 carbon of corticosteroid is influenced by C17 and C21 substitution, and its restricted rotation is controlled by intramolecular forces, not by crystal packing forces.^43^ NMR relaxometry and CSA tensor offer a more complete investigation of the molecular dynamics at various carbon nuclei sites of this corticosteroid. It is noticeable from Tables 2 and 3 that the CSA parameters and spin–lattice relaxation time of C21 and C22 nuclei reside on the side-chain of dexamethasone are substantially large. The spin–lattice relaxation time of C22 is 231 s and it is 190 s for C21 nuclei. The molecular correlation time is 4.5 × 10^−4^ s for C21 and 5.7 × 10^−5^ s for C22. These imply that the rotational motion of the side-chain of dexamethasone is restricted. The magnetization decay curves at numerous carbon sites of dexamethasone is shown in Fig. 6(a–d), and the bar-diagram of spin-lattice relaxation time at chemically different carbon sites is shown in Fig. 6(e).

**Table 3:** Spin–lattice relaxation time and molecular correlation time at various carbon nuclei sites of dexamethasone

Carbon atom	Spin–lattice relaxation time (s)	Molecular correlation time (s)
C21	190 ± 10	4.5 × 10^−4^
C3	190 ± 10	4.4 × 10^−4^
C5	192 ± 10	7.3 × 10^−4^
C1	221 ± 10	7.3 × 10^−4^
C2	235 ± 10	4.5 × 10^−4^
C4	233 ± 10	3.2 × 10^−4^
C9	201 ± 10	5 × 10^−5^
C17	215 ± 10	5 × 10^−6^
C16	321 ± 10	5 × 10^−5^
C22	231 ± 10	5.7 × 10^−5^
C13	135 ± 5	3 × 10^−5^
C10	175 ± 5	1.7 × 10^−5^
C14	310 ± 10	1 × 10^−4^
C12	270 ± 10	1 × 10^−5^
C11	188 ± 10	1.8 × 10^−5^
C8	268 ± 10	3.6 × 10^−5^
C15	252 ± 10	2.5 × 10^−5^
C7	300 ± 10	1.3 × 10^−5^
C6	300 ± 10	2.1 × 10^−5^
C19	70 ± 5	5.1 × 10^−6^
C18	50 ± 5	1.3 × 10^−6^
C20	60 ± 5	2.1 × 10^−6^

For ^13^C nucleus, the relaxation mechanism is governed by the chemical shift anisotropy interaction and the hetero-nuclear dipole–dipole coupling interaction. The role of chemical shift anisotropy interaction in ^13^C spin–lattice relaxation mechanism is expressed as^50–53^2
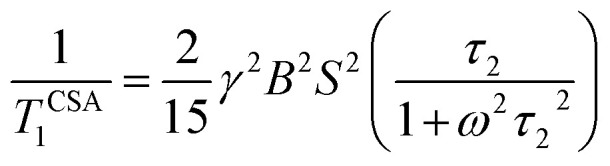
where correlation time *τ*_c_ = 3*τ*_2_ and *B* is the applied magnetic field. Where *S*^2^ = (Δ*δ*)^2^(1 + *η*^2^/3) and 
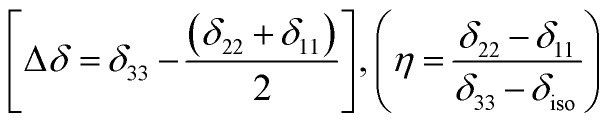
.

The role of heteronuclear dipole–dipole coupling on spin–lattice relaxation mechanism is expressed as^52^3
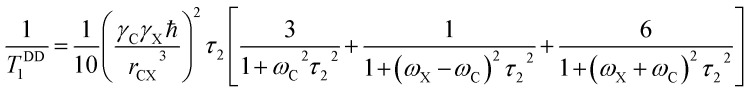


By keeping only the first term,4
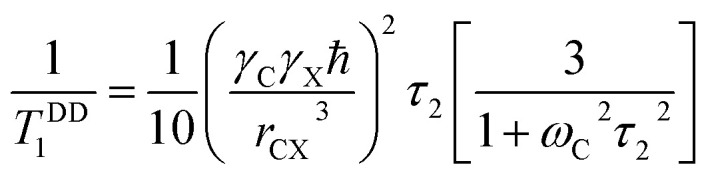
where X represent hydrogen, oxygen and nitrogen atoms. *r*_CX_ is the distance between carbon and neighbouring atoms hydrogen, oxygen, fluorine, which is determined by X-ray crystal structural studies.^49^ Larmour precession frequency *ω* = 2π*f* = 2 × 3.14 × 125.758 MHz = 789.76024 MHz; *B* = 11.74 T, *γ*_C_ = 10.7084 MHz T^−1^, *γ*_H_ = 42.577 MHz T^−1^, *ℏ* = 1.054 × 10^−34^ J s.

The spin–lattice relaxation rate for ^13^C can be articulated as5
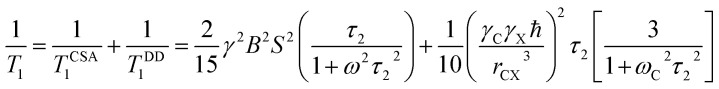


**Fig. 7:**
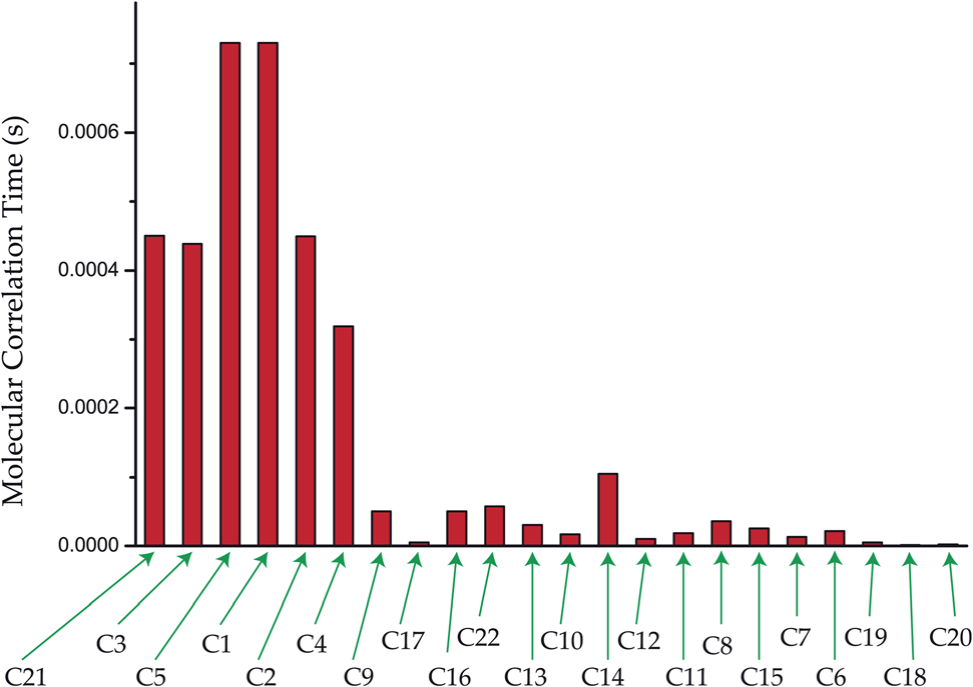
Molecular correlation time of dexamethasone at crystallographically different sites.

Molecular correlation time is calculated by this equation (Fig. 7). The bar-diagram of molecular correlation time at crystallographically different carbon nuclei sites of dexamethasone is shown in Fig. 7.

The molecular correlation time of all the atoms except C10 is of the order of 10^−4^ s. Molecular correlation time of methyl group carbon C18, C19, and C20 is of the order of 10^−6^ s. As C19 is bonded with C10, hence the molecular correlation time is reduced to the order of 10^−5^ s. Molecular correlation time of all the atoms except C14 of ‘B’, ‘C’ and ‘D’ rings is of the order of 10^−5^ s. The difference in molecular correlation time in various regions of the molecule demonstrates the existence of different degrees of freedom within the molecule. This may be the reason of the various biological activities exhibited by the molecule.

## 4. Conclusion

The corticosteroid molecule is associated with flexible unsaturated rings, side chains and, substituents. The flexibility and conformation of rings and side-chain are highly influenced by these substituents of corticosteroid. The molecular packing patterns are determined by hydrogen-bonding, ring conformation, and side-chain orientation. The structure and dynamics of corticosteroid dexamethasone are determined by measuring CSA parameters, site-specific spin–lattice relaxation time, and molecular correlation time at twenty-two crystallographically different carbon sites. ‘A’ ring is the area of the conformational flexibility of the corticosteroid molecule. The CSA parameters of all carbon nuclei reside on ‘A’ ring except C10 are substantially large. C10 is in sp^3^ hybridization, which is the reason of the lower values of CSA parameters. The double bonds between C1 and C2 and C4 and C5 of the six membered ‘A’ ring increased the anti-inflammatory activity and diminished the salt-retaining properties of dexamethasone. The presence of π-electrons is the reason of the higher values of the CSA parameters of C1, C2, C4 and C5 carbon nuclei compared to the carbon nuclei reside on ‘B’, ‘C’, and ‘D’ rings. The existence of the fluorine atom, bonded with C9 carbon atom, increased binding affinity of the corticosteroid receptor and retarded oxidation of the hydroxyl group attached with C11 atom. The anti-inflammatory assays and glycogen deposition rates are enhanced by the presence of fluorine atom. In general, the biological activity of corticosteroid is proportionally increased with the electronegativity of the C9 substituent. The CSA parameters of C9 nuclei are higher than C6, C7, C18, and C10 nuclei reside on the ‘B’ ring. The electronegative fluorine atom attracts the electron cloud surrounding C9 nuclei. As a consequence, the influence of the nuclear shielding effect is lowered and the effective magnetic field experienced by the nucleus is increased, which leads to the higher values of the CSA parameters. The molecular correlation time of C1, C2, C3, C4, C5 nuclei is of the order of 10^−4^ s and it is of the order of 10^−5^ s for C10. The spin–lattice relaxation time of all the carbon nuclei resides on ‘A’, ‘B’, ‘C’, and ‘D’ rings and side-chain of dexamethasone is quite large. The directionality of hydrogen bond donors and acceptors emerge a fascinating crystallographic pattern for corticosteroid. The crystal packing arrangement is controlled by intermolecular and intramolecular forces. These are the reason for the large values of spin–lattice relaxation time. The crystallographic findings demonstrate that the orientation of the side-chain attached with C17 carbon of corticosteroid is influenced by C17 and C21 substitution. The rotation is controlled by intramolecular forces, not by crystal packing forces.^43^ NMR relaxometry and CSA tensor offer a more complete investigation of the molecular dynamics at various nuclei sites of this corticosteroid. It is noticeable from Tables 2 and 3 that the CSA parameters and spin–lattice relaxation time of C21 and C22 nuclei reside on the side-chain of dexamethasone are substantially large. The spin–lattice relaxation time of C22 is 231 s and it is 190 s for C21 nuclei. The molecular correlation time is 4.5 × 10^−4^ s for C21 and 5.7 × 10^−5^ s for C22. These imply that the rotational motion of the side-chain of dexamethasone is restricted. The difference in molecular correlation time at various regions of the molecule demonstrates the existence of different degrees of freedom within the molecule. This may be the reason of the various biological activities exhibited by the molecule. The spin–lattice relaxation time and the molecular correlation time of the methyl groups are the lowest among all the atoms of the dexamethasone, these methyl groups increase the potency and duration of the drug actions. These types of elaborative studies of the structure and dynamics of an important corticosteroid with multiple biological activities are necessary to develop the advanced medicine and it will help to understand the structure–activity relationships of corticosteroid.

## Conflicts of interest

There are no conflicts to declare.

## Supplementary Material
